# Supplementary material for: Near telomere-to-telomere genome assemblies of two Chlorella species unveil the composition and evolution of centromeres in green algae
Source: BMC Genomics. 2024 Apr 10;25:356. doi: 10.1186/s12864-024-10280-8 (PMC11005252; doi:10.1186/s12864-024-10280-8)
Supplement: Supplementary file 1 — Supplementary Material 1. [file 12864_2024_10280_MOESM1_ESM.pdf]

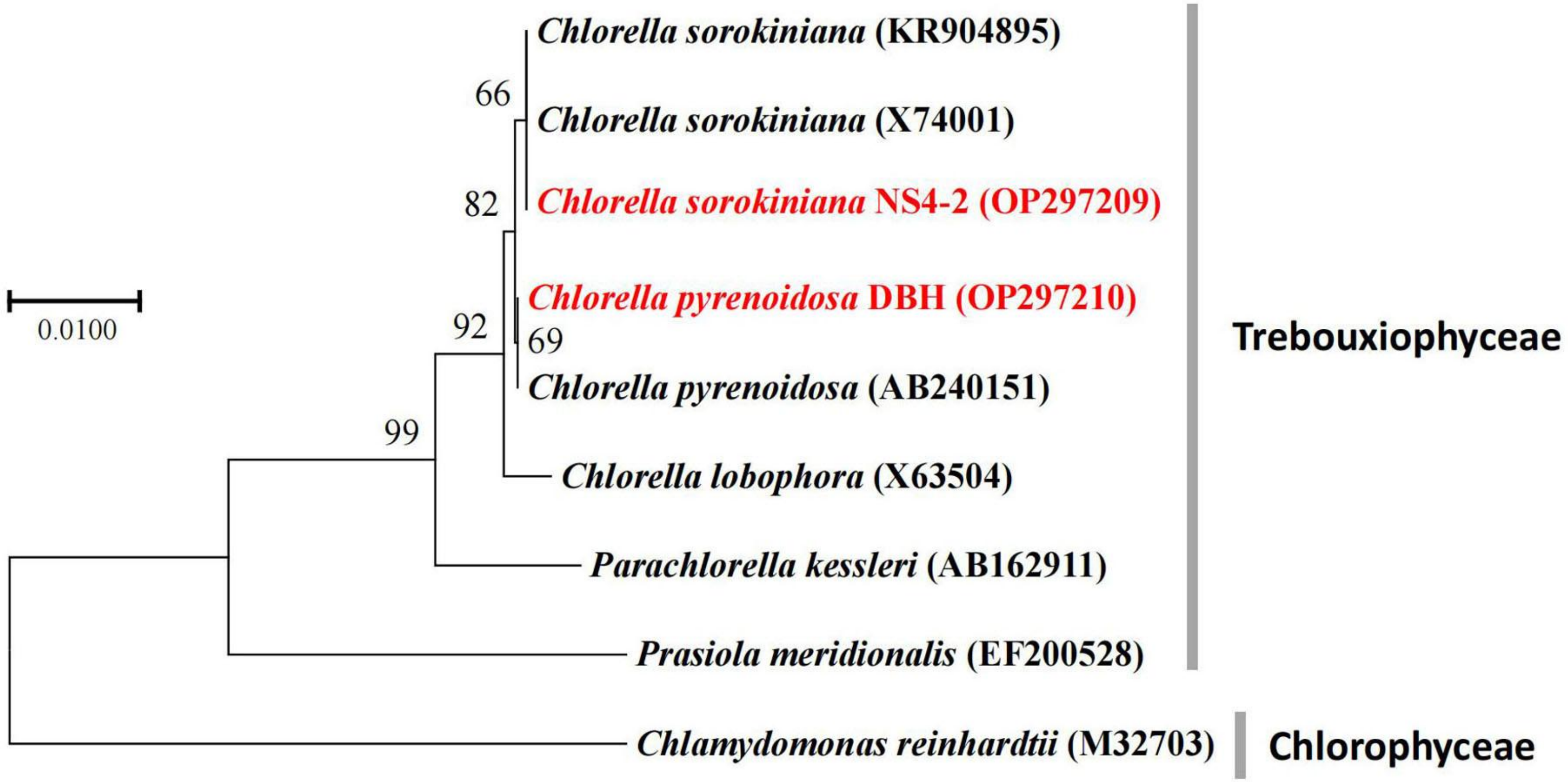

Supplementary Fig. S1. Maximum likelihood phylogenetic tree based on the analysis of 18S gene sequences. GenBank accession numbers in brackets.

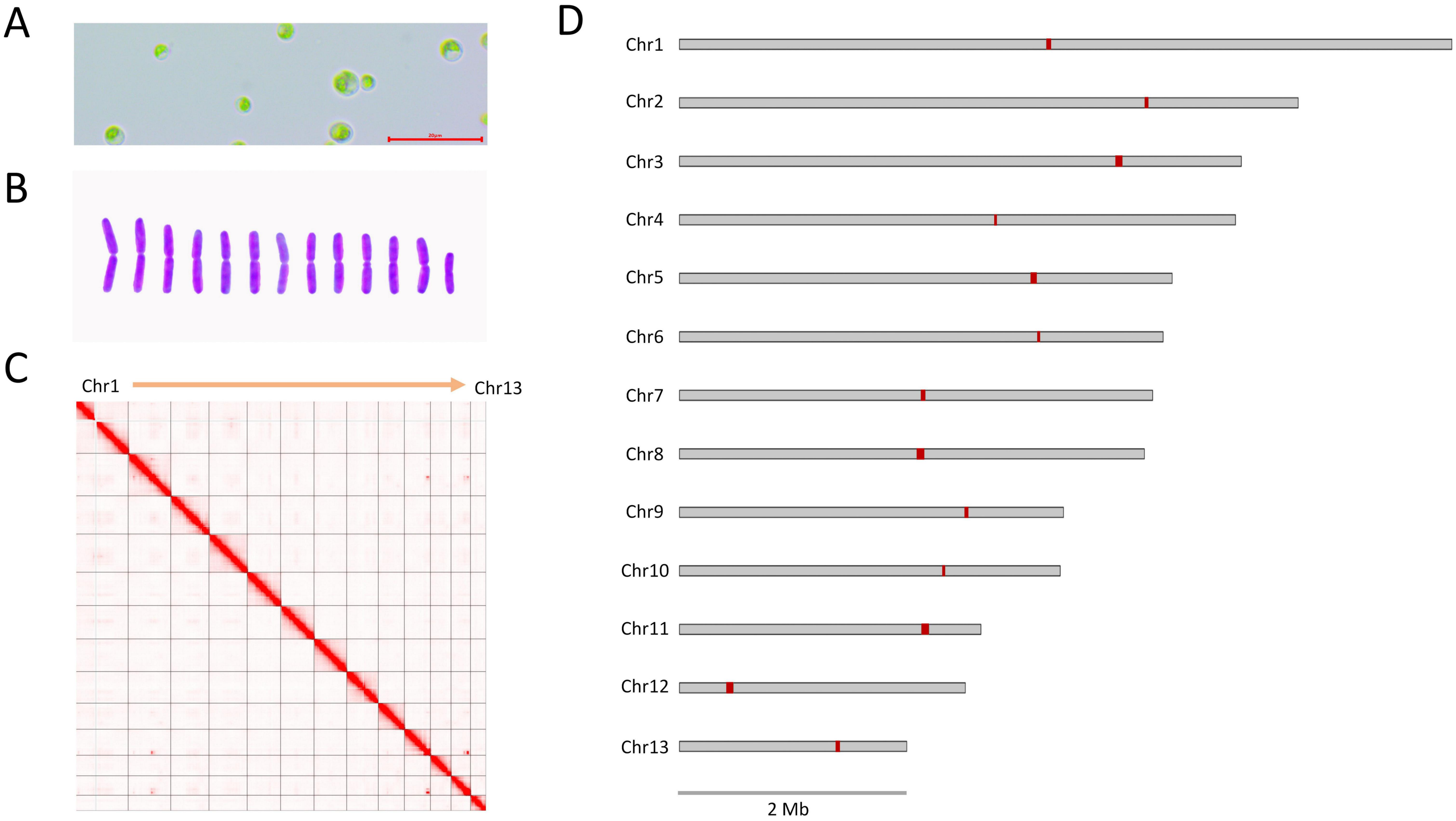

Supplementary Fig. S2. Near telomere-to-telomere genome assemblies and centromere identification of *Chlorella pyrenoidosa* strain DBH. **(A)** Micrograph of *C. pyrenoidosa* DBH, bar = 20 µm; **(B)** The karyotype diagram indicates that *C. pyrenoidosa* DBH has 13 chromosomes; **(C)** Hi-C contact map of the *C. pyrenoidosa* DBH T2T genome assembly (chromosome 1-13); **(D)** Schematic overview of the chromosomes of *C. pyrenoidosa* DBH showing the predicted centromere regions (red bands).

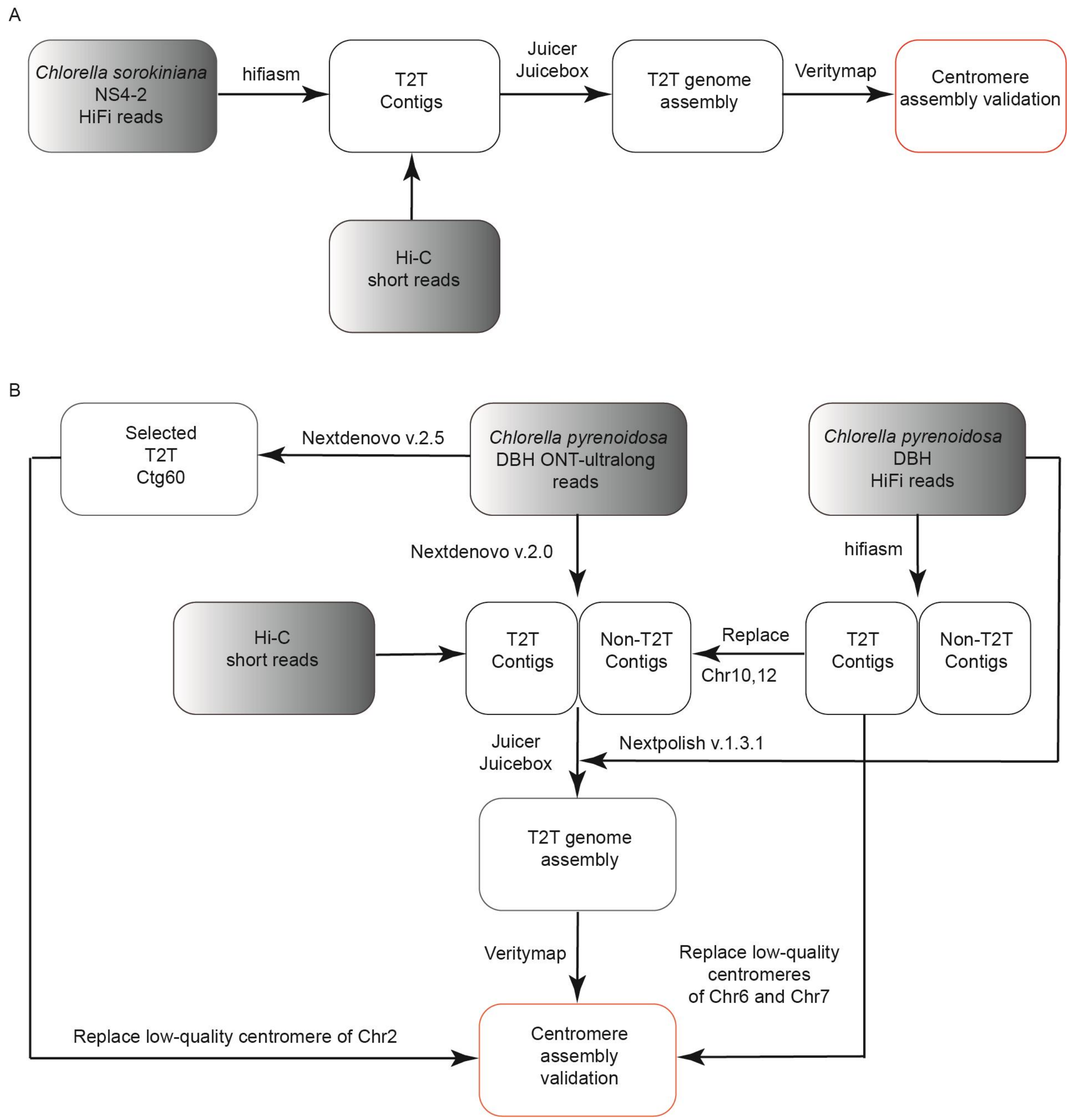

Supplementary Fig. S3. Overview of the telomere-to-telomere genome assembly pipelines. **(A)** *Chlorella sorokiniana* NS4-2 genome assembly; **(B)** *Chlorella pyrenoidosa* DBH genome assembly.

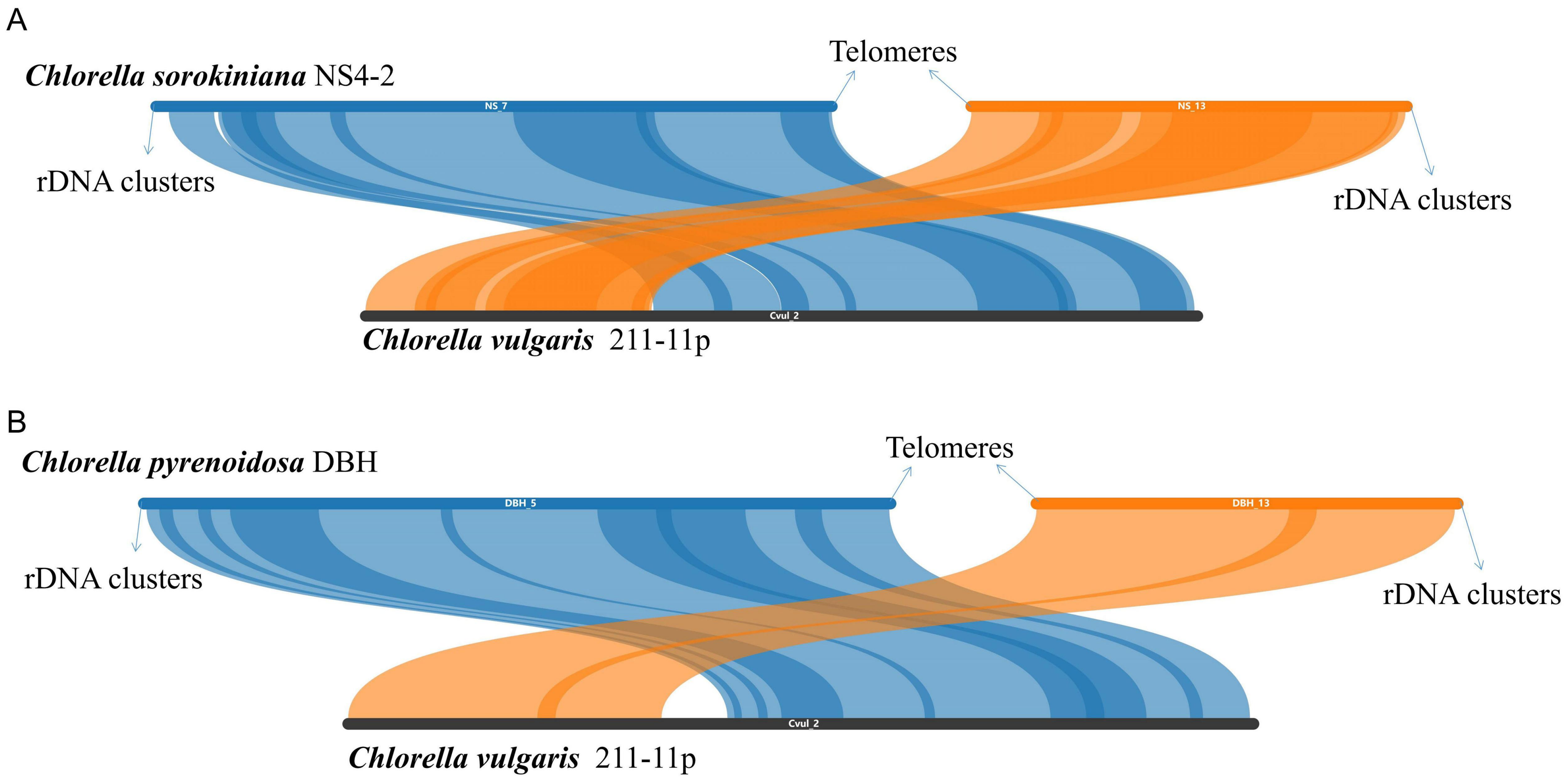

Supplementary Fig. S4. Illustration of collinearity between chromosomes. (A) Collinearity between two contigs of *Chlorella sorokiniana* NS4-2 and chromosome 2 of *Chlorella vulgaris*; (B) Collinearity between two contigs of *Chlorella pyrenoidosa* DBH and chromosome 2 of *Chlorella vulgaris*.

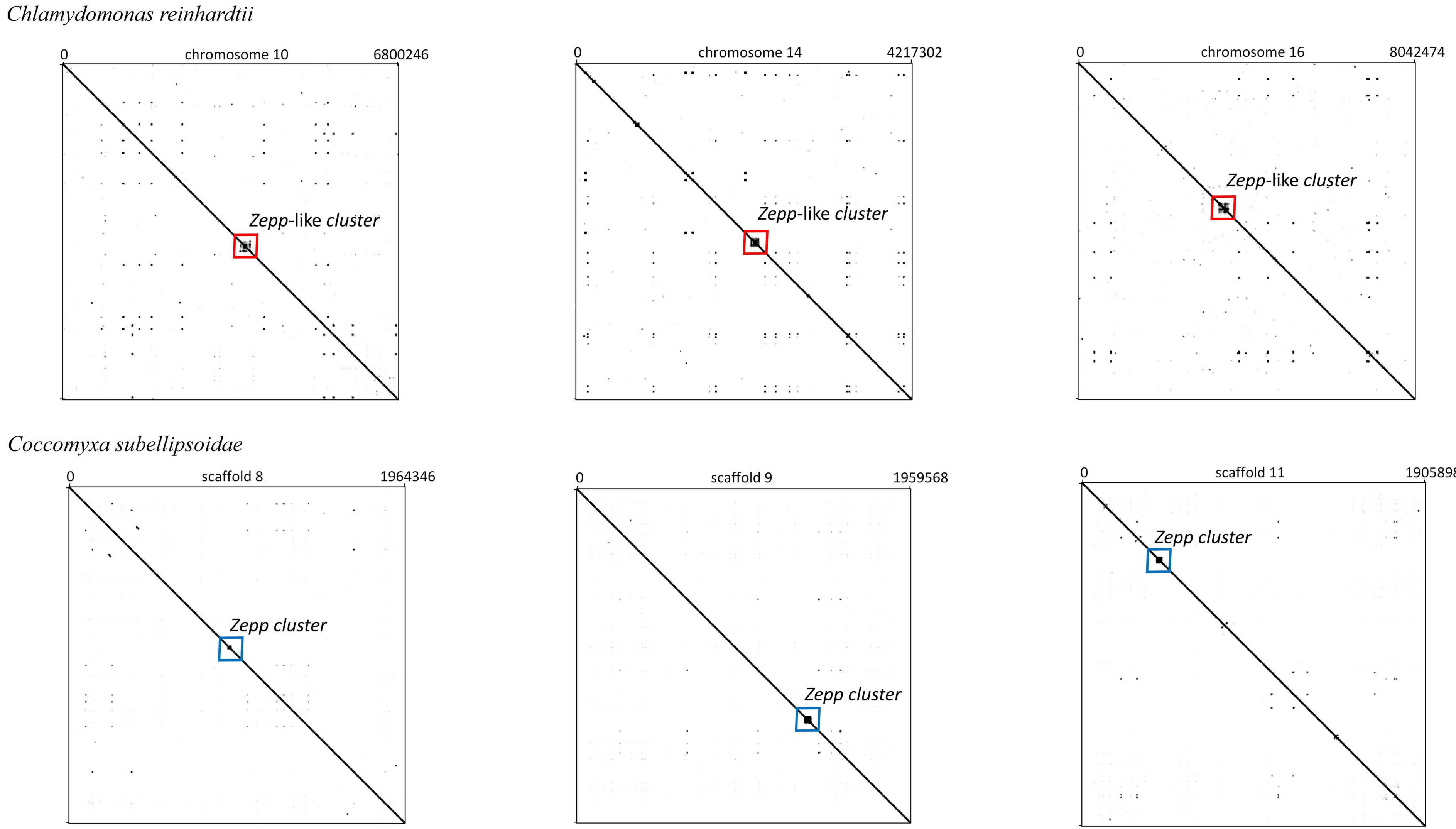

Supplementary Fig. S5. Self-dot plot showing potential centromeric features and regions of two previous assemblies, *Chlamydomonas reinhardtii* and *Coccomyxa subellipsoidae*.

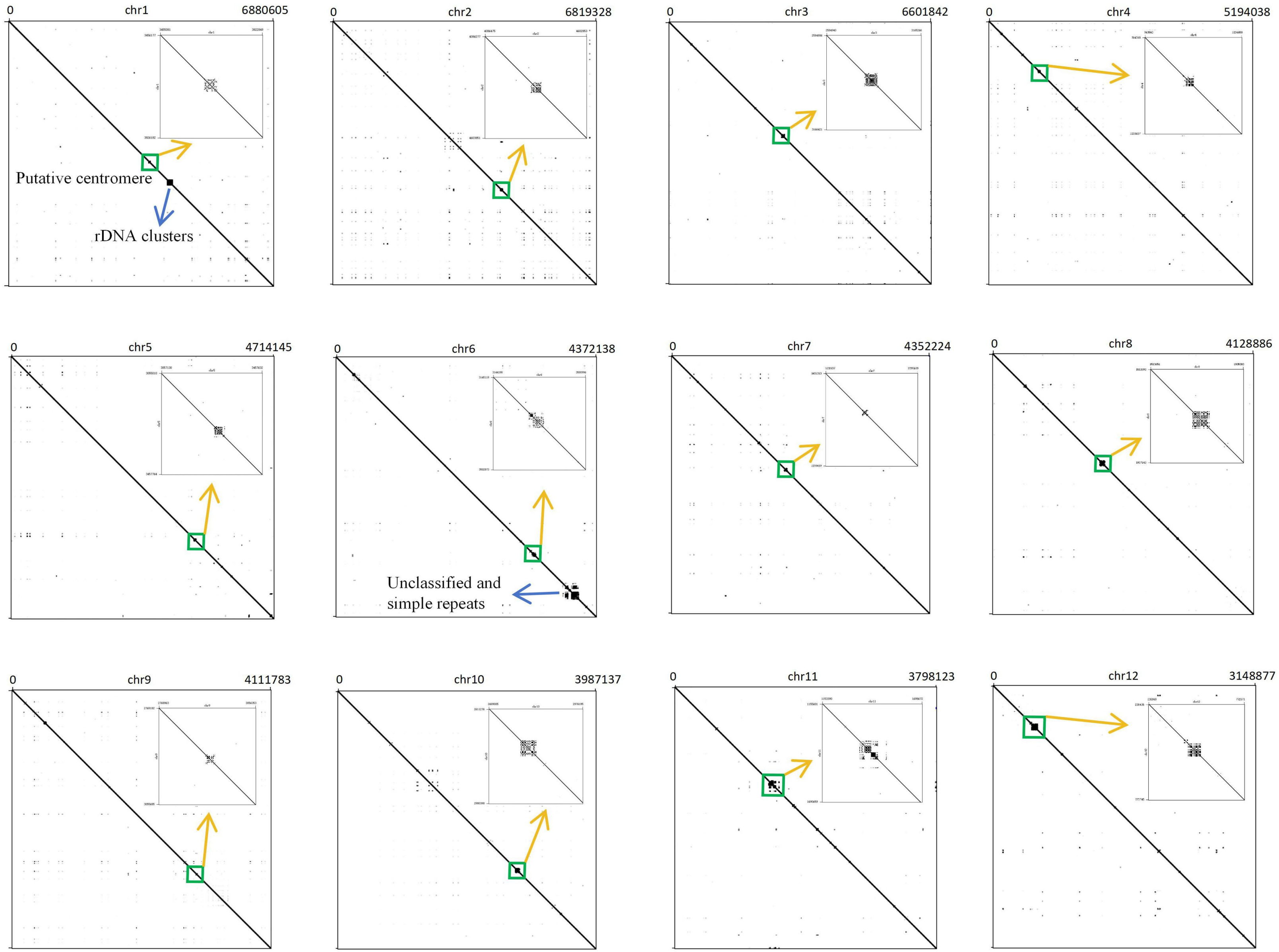

Supplementary Fig. S6. Self-dot plot of the 12 chromosomes of *Chlorella sorokiniana* NS4-2, with green boxes indicating potential centromeric regions.

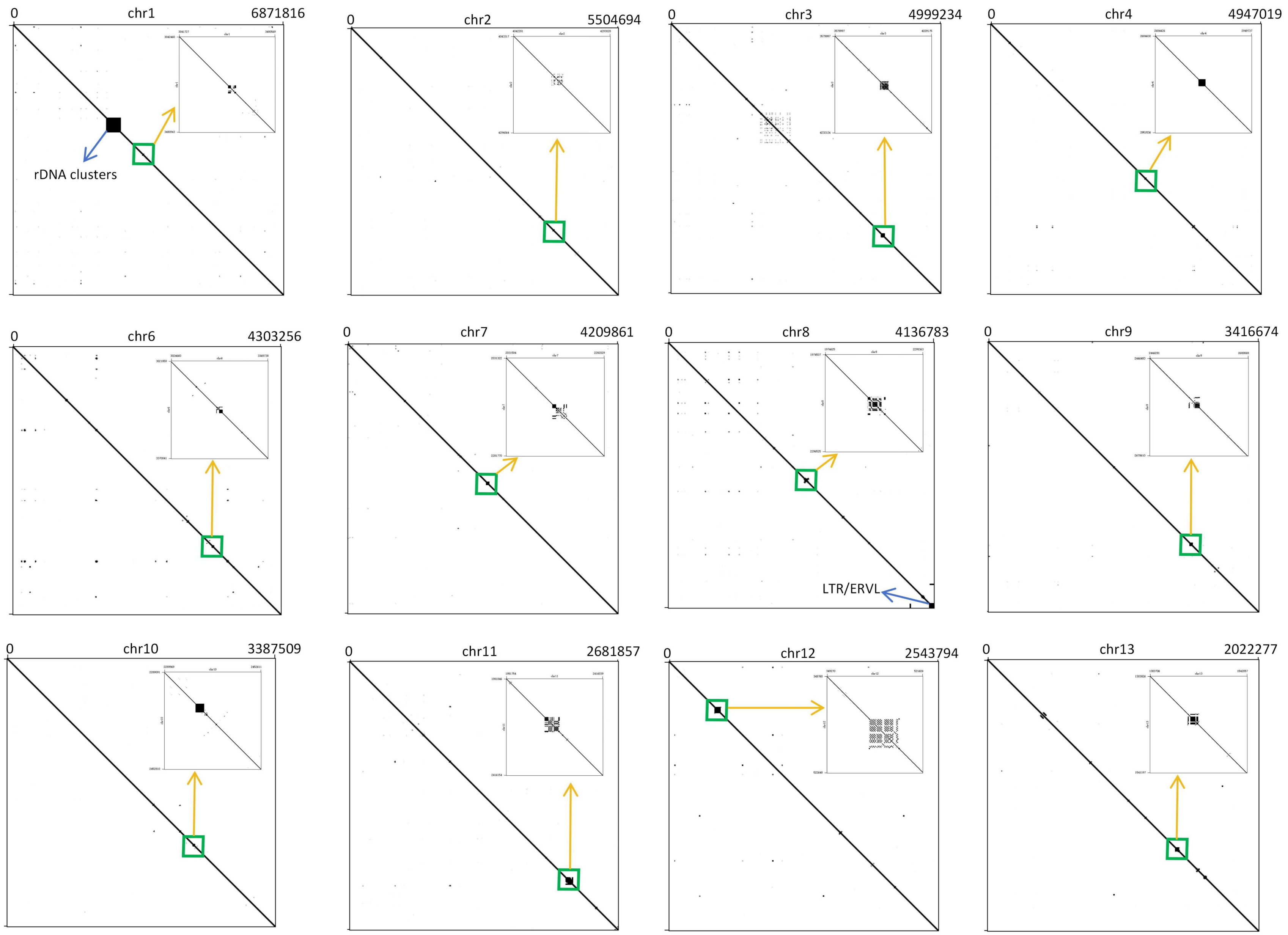

Supplementary Fig. S7. Self-dot plot of the 13 chromosomes of *Chlorella pyrenoidosa* DBH, with green boxes indicating potential centromeric regions.

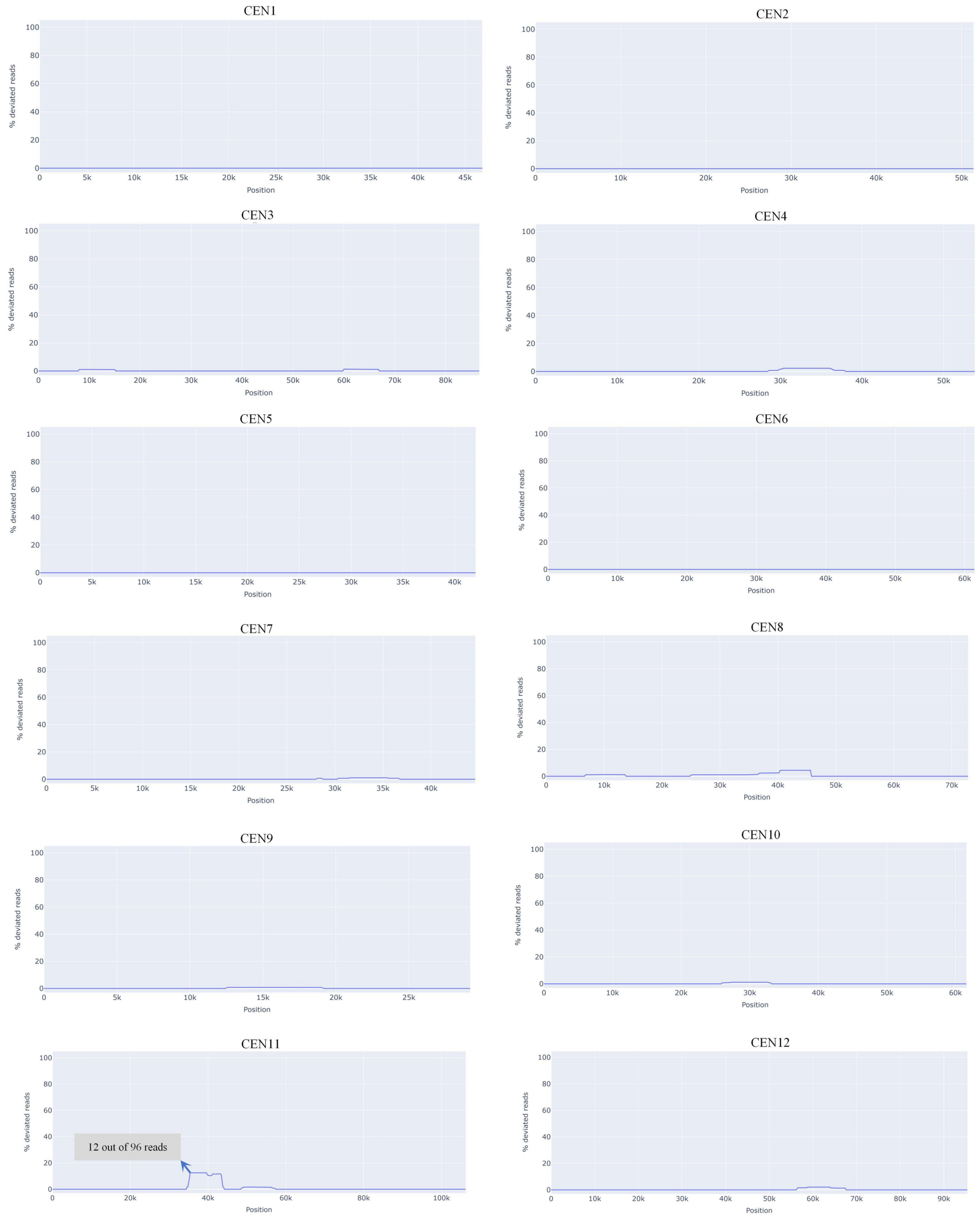

Supplementary Fig. S8. Evaluation of the 12 putative centromeric assemblies of *Chlorella sorokiniana* NS4-2 using VerityMap. The x-axis represents the position of the centromere, while the y-axis indicates the percentage of deviated reads, limited or negligible in each case. At most, only a low percentage (12.5%) of deviated reads were detected in the centromere of chromosome 11 (CEN11), which we interpret to mean there were no potentially large structural errors in the assembly.

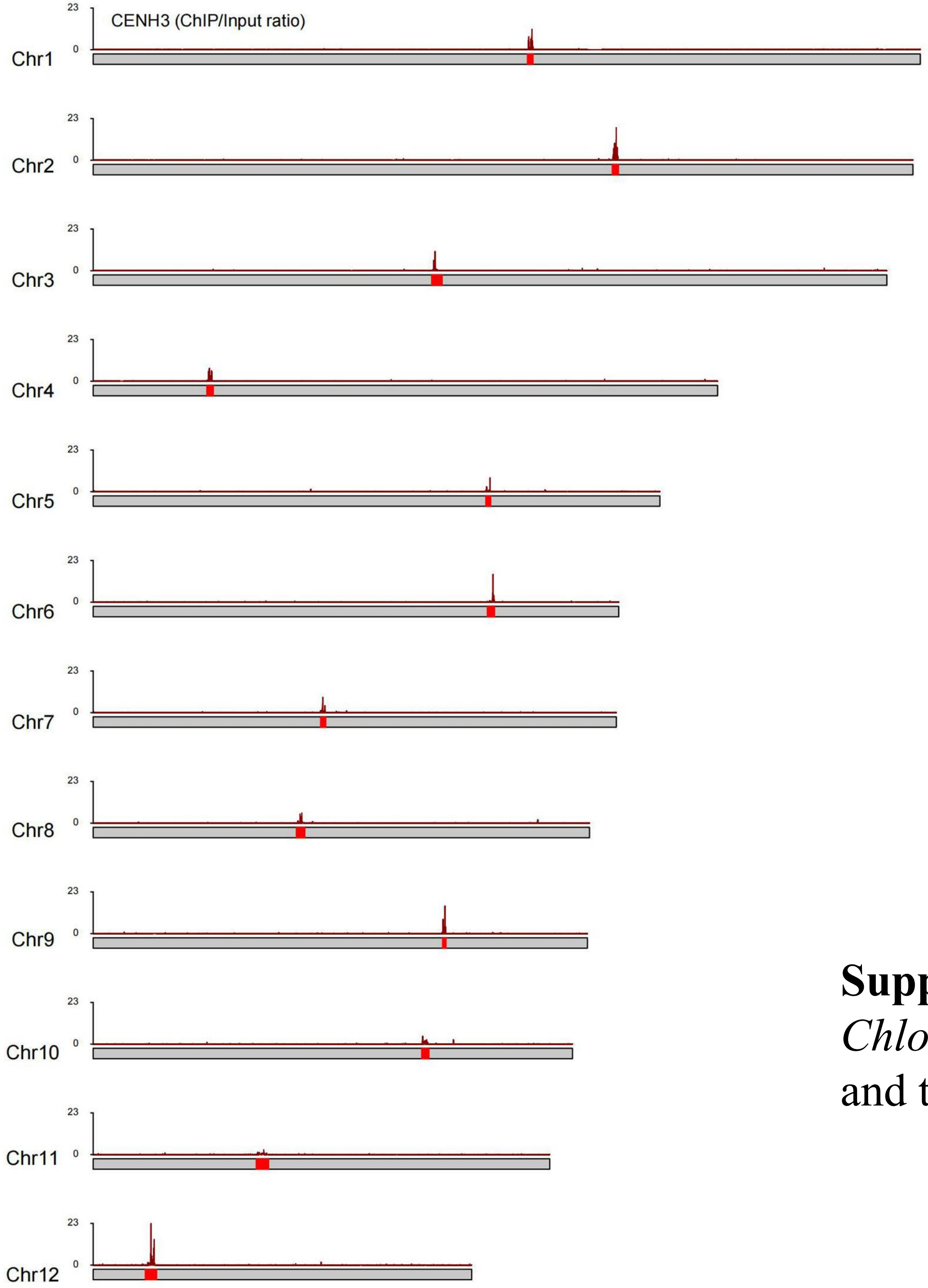

Supplementary Fig. S9. ChIP/Input ratio of the 12 chromosomes of *Chlorella sorokiniana* NS4-2. Schematic overview showing the ratio and the putative centromere regions (red bands).

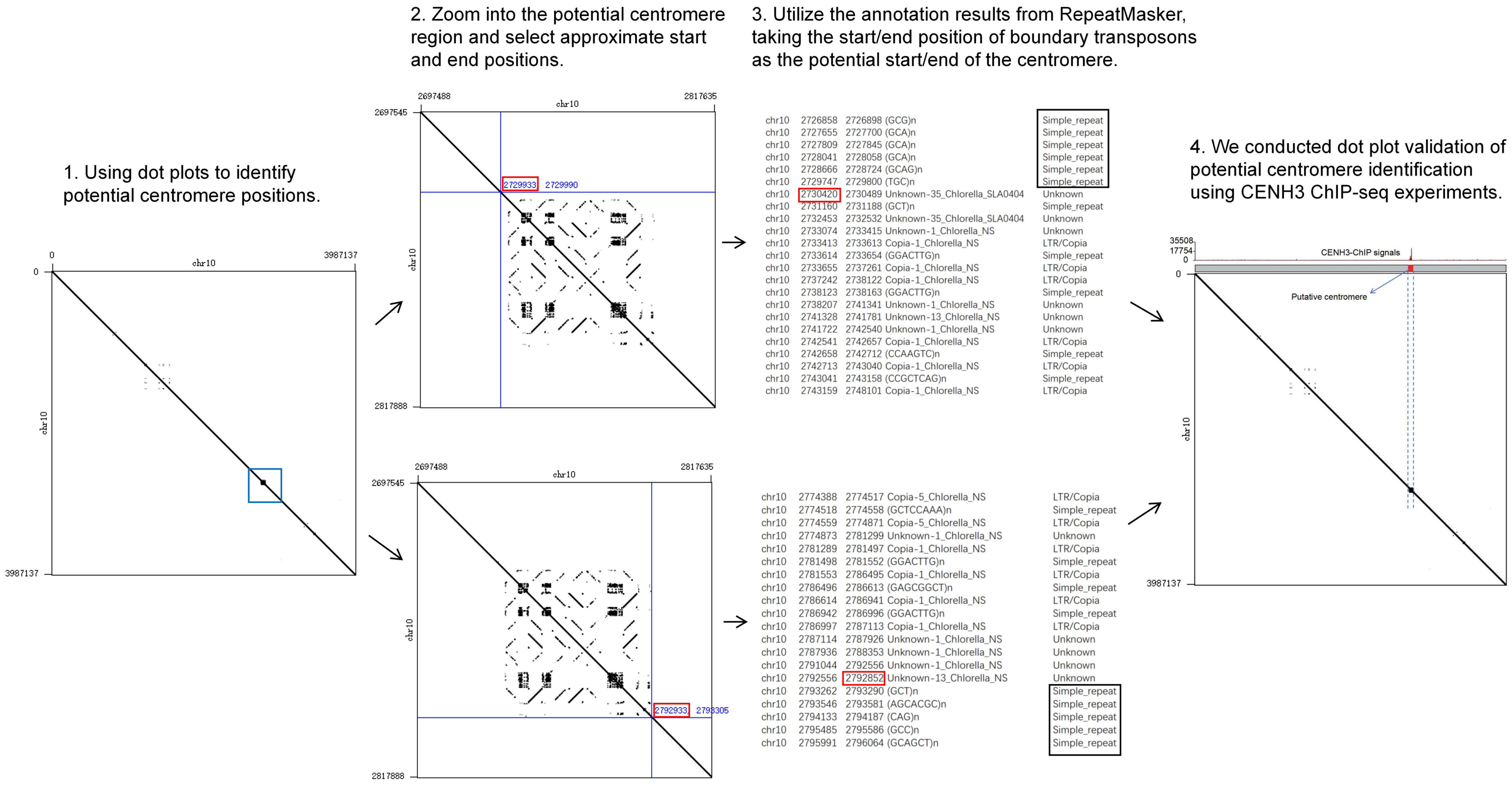

Supplementary Fig. S10. Overview of the dot plot method of identifying potential centromere positions in the genome, illustrated using chromosome 10 of *Chlorella sorokiniana* NS4-2.

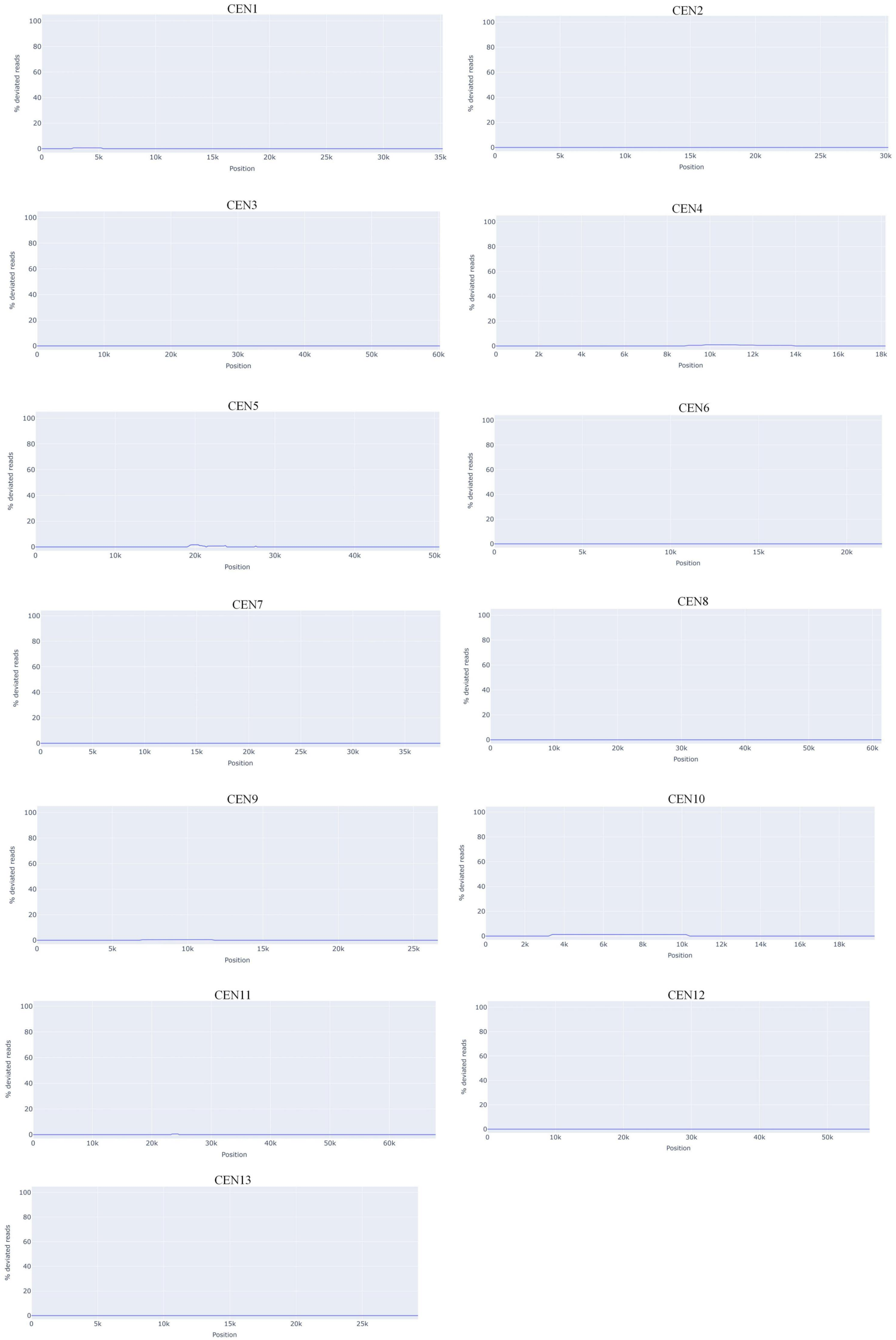

Supplementary Fig. S11. Evaluation of the 13 putative centromere assemblies of *Chlorella pyrenoidosa* DBH using VerityMap. The x-axis represents the position of the centromere, while the y-axis indicates the percentage of deviated reads (negligible in all cases).

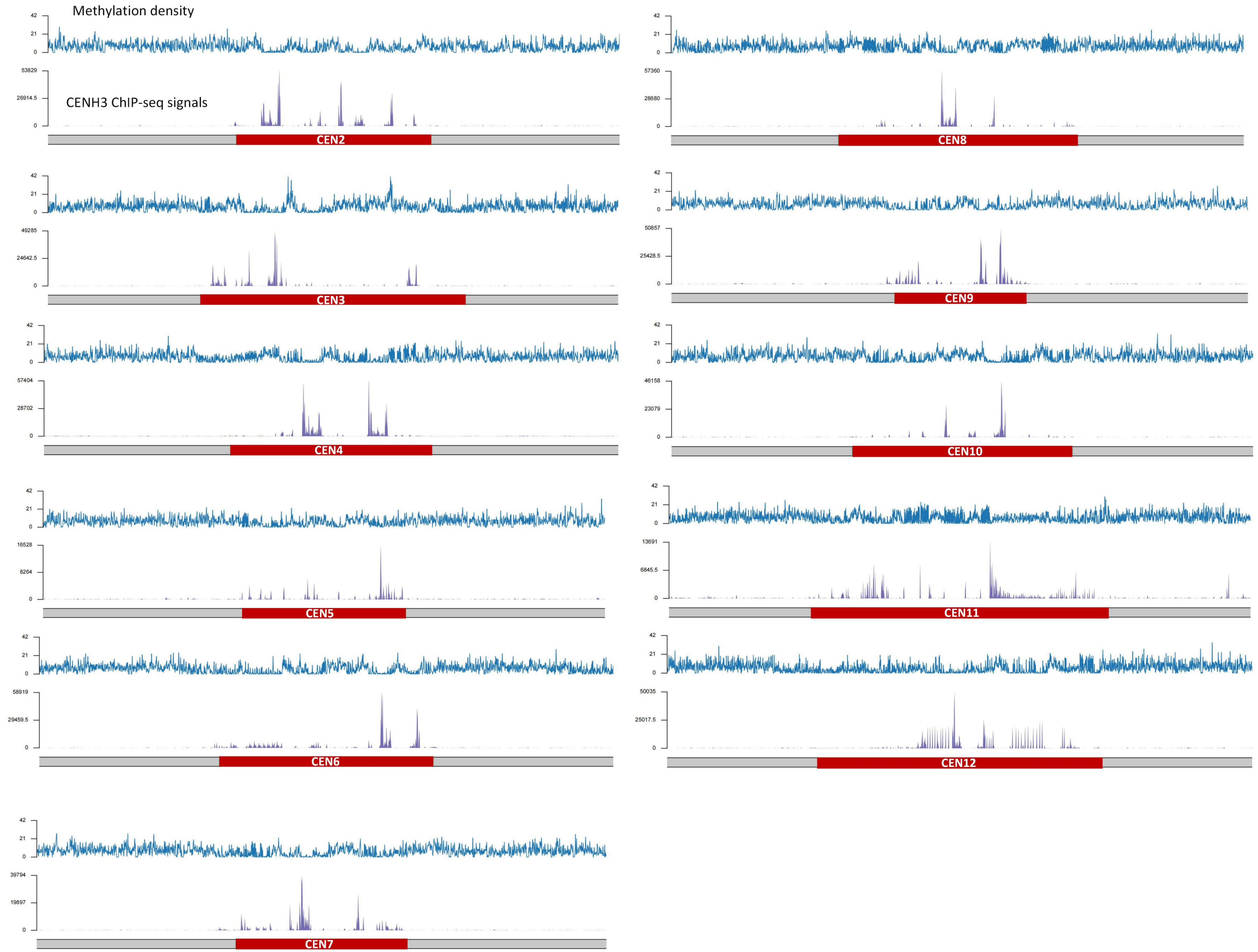

Supplementary Fig. S12. Centromeric methylation features of *Chlorella sorokiniana* NS4-2. Schematic overview depicting each centromere along with its 50 Kb upstream and downstream regions of *C. sorokiniana* NS4-2. The illustration shows the distribution of methylation density (determined by HiFi data), CENH3 ChIP-seq signals, and the location of centromeric regions (red bands).

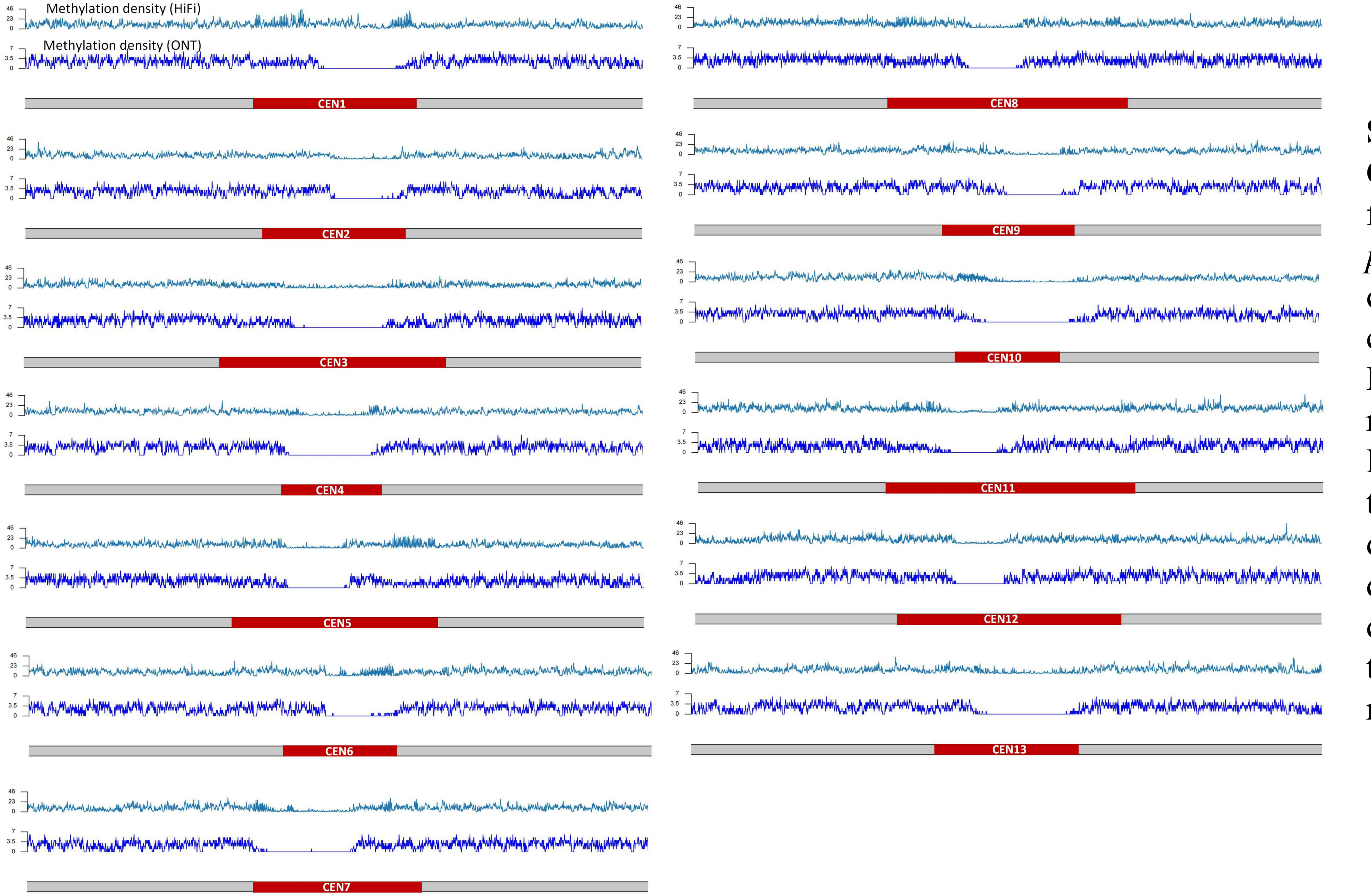

Supplementary Fig. S13. Centromeric methylation features of *Chlorella pyrenoidosa* DBH. Schematic overview depicting each centromere along with its 50 Kb upstream and downstream regions of *C. pyrenoidosa* DBH. The illustration shows the distribution of methylation density determined by HiFi data, methylation density determined by ONT data, and the loction of centromeric regions (red bands).

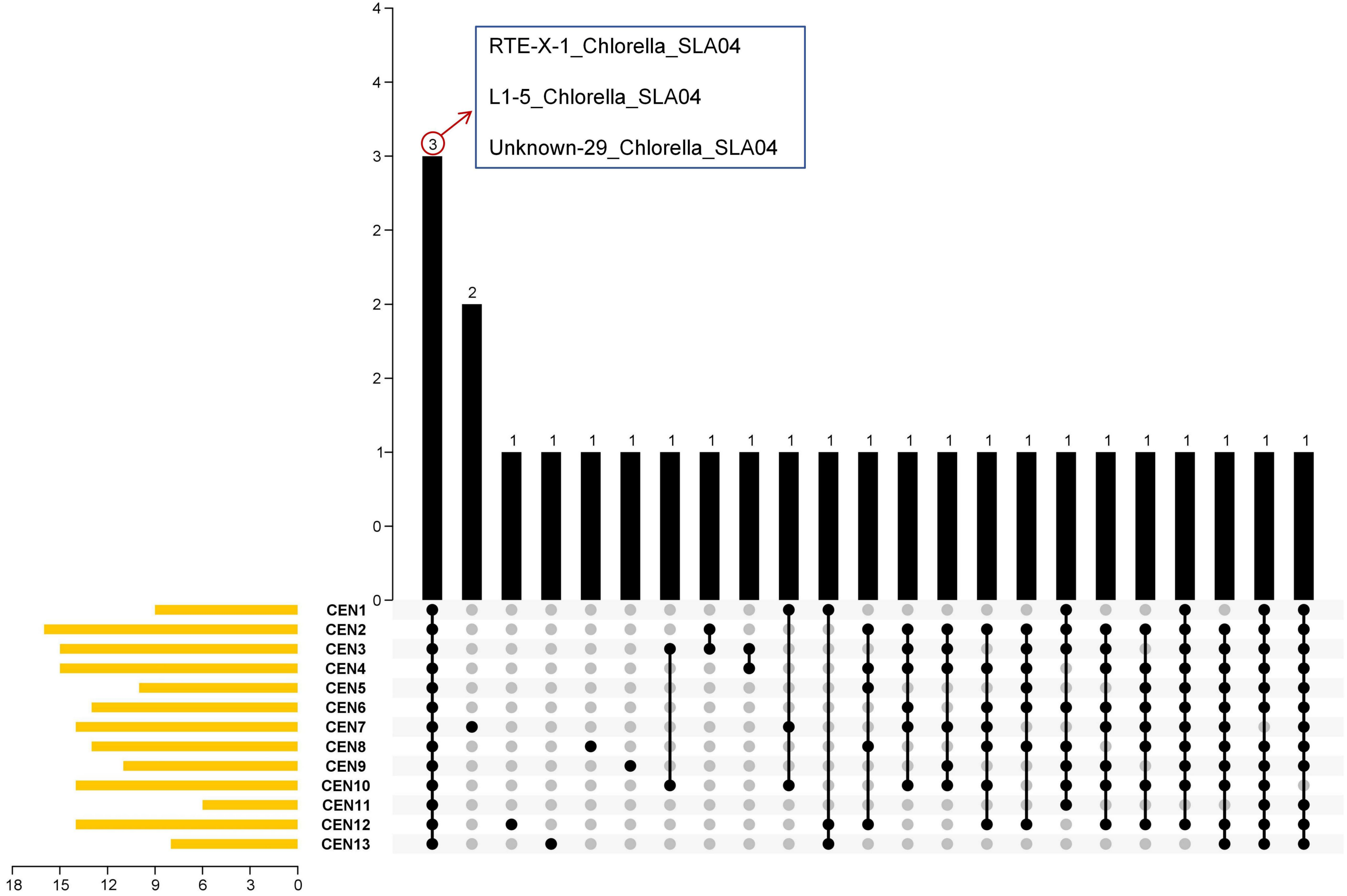

Supplementary Fig. S14. Upset plot depicting the overlap of centromeric repeat subfamilies among 13 centromeres (CENs) in *Chlorella* sp. SLA-04.

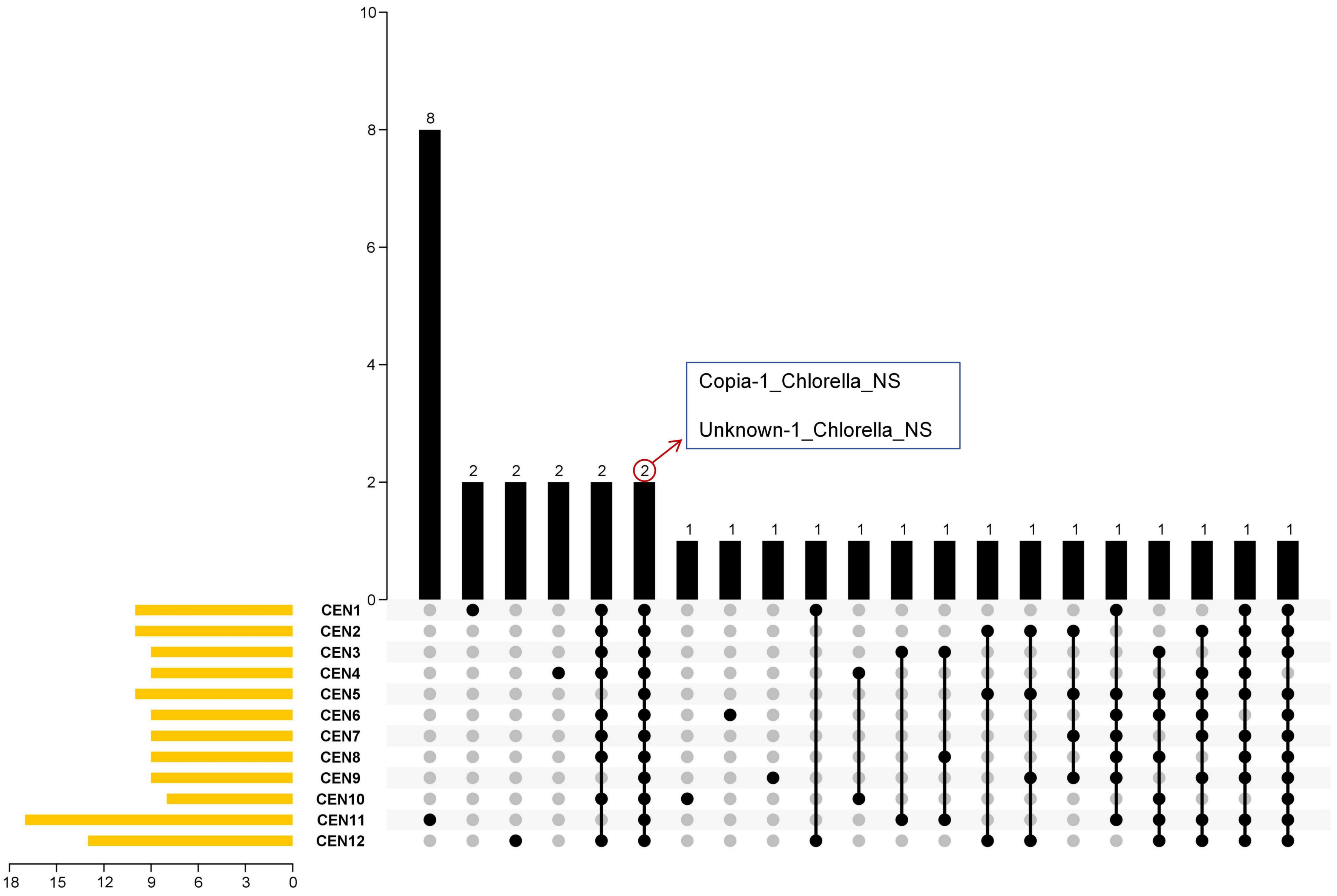

Supplementary Fig. S15. Upset plot depicting the overlap of centromeric repeat subfamilies among 12 centromeres (CENs) in *Chlorella sorokiniana* NS4-2.

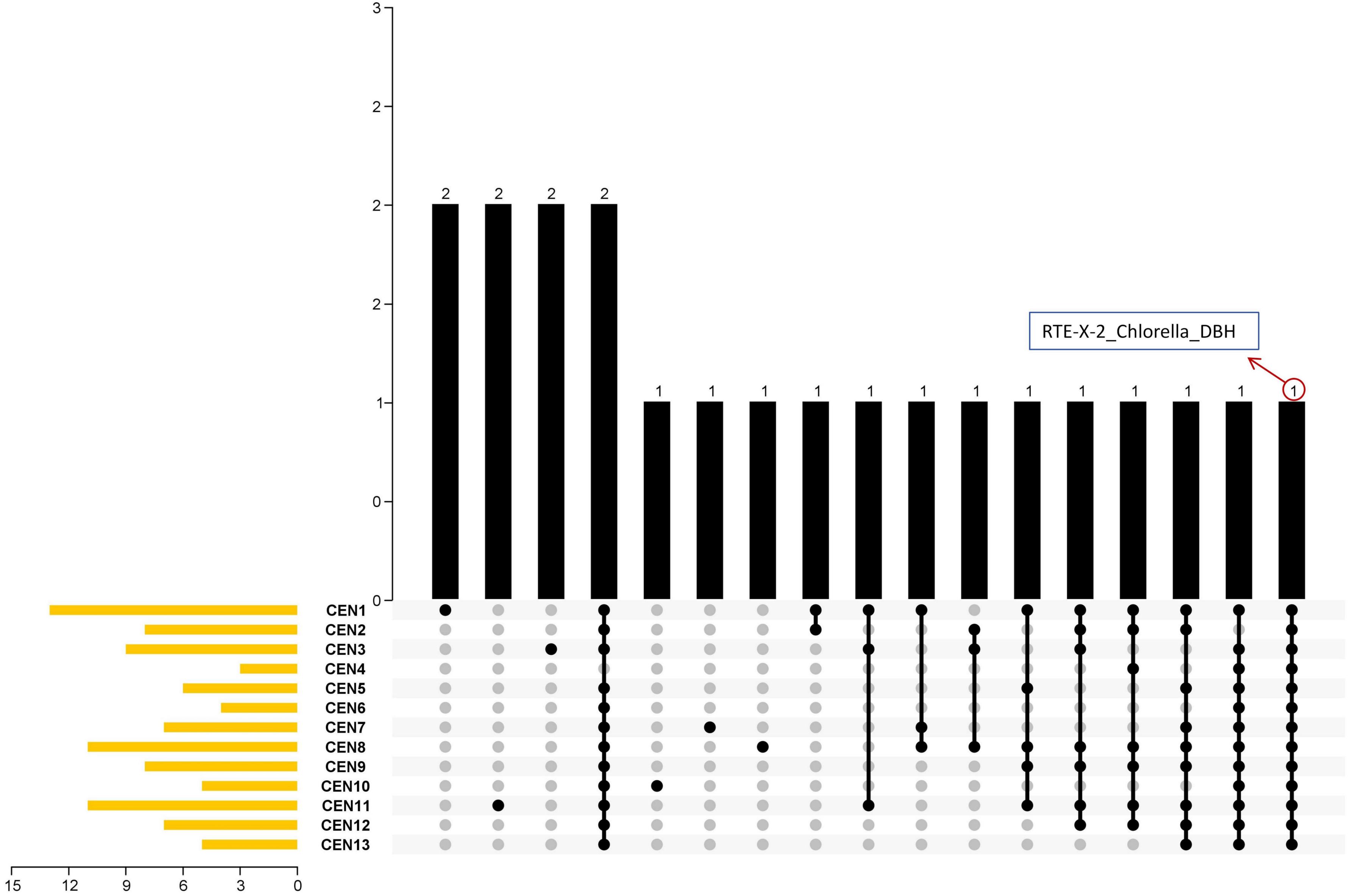

Supplementary Fig. S16. Upset plot depicting the overlap of centromeric repeat subfamilies among 13 centromeres (CENs) in *Chlorella pyrenoidosa* DBH.

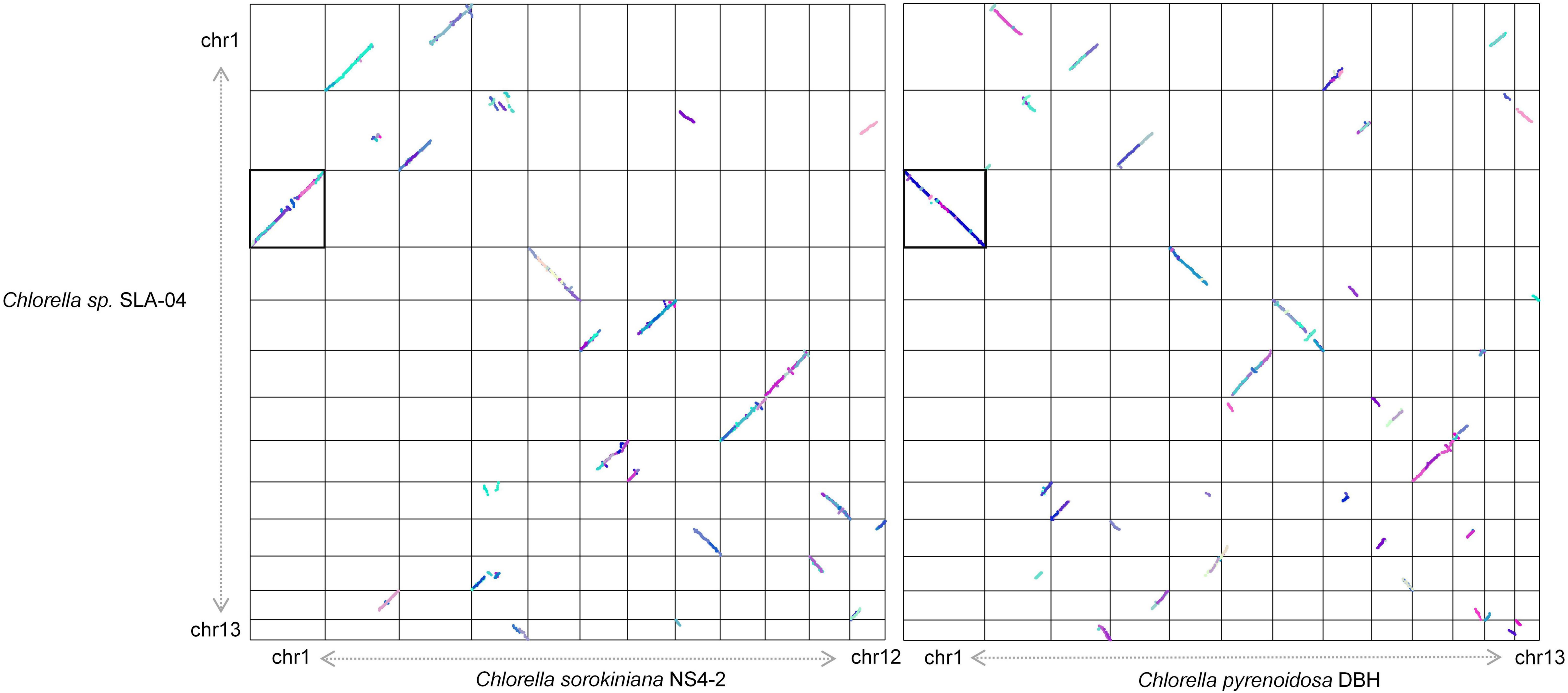

Supplementary Fig. S17. Dot plots of interspecies syntenic blocks of *Chlorella* sp. SLA-04, *Chlorella sorokiniana* NS4-2 and *Chlorella pyrenoidosa* DBH. Each dot indicates a syntenic gene pair detected by MCScanX. Synteny blocks were colored by MCScanX.
